# Supplementary material for: Size Scaling of the Electrochemical Performance of Ti3C2T x MXene Microelectrode Arrays for Electrophysiological Recording and Stimulation
Source: Small. 2026 May 12;22(36):e14955. doi: 10.1002/smll.202514955 (PMC13175084; doi:10.1002/smll.202514955)
Supplement: Supplementary file 1 — Supporting File: smll73732‐sup‐0001‐SuppMat.pdf. [file SMLL-22-e14955-s001.pdf]

Supporting Information

Size Scaling of the Electrochemical Performance of $Ti_3C_2T_x$ MXene Microelectrode Arrays for Electrophysiological Recording and Stimulation

*Spencer R. Averbeck[1,2], Raghav Garg[2,3], Royce Dong[1,2], Daryl Hurwitz[2], Nicholas V. Apollo[4], Michael S. Beauchamp[4], Flavia Vitale[1,2,3,5,6]**

[1] S. R. Averbeck, R. Dong, F. Vitale
Department of Bioengineering,
University of Pennsylvania,
Philadelphia, Pennsylvania – 19104, USA.
E-mail: vitalef@pennmedicine.upenn.edu

[2] S. R. Averbeck, R. Garg, R. Dong, D. Hurwitz, N.V. Apollo, F. Vitale
Center for Neuroengineering and Therapeutics,
University of Pennsylvania,
Philadelphia, Pennsylvania – 19104, USA.

[3] R. Garg, F. Vitale
Department of Neurology,
University of Pennsylvania,
Philadelphia, Pennsylvania – 19104, USA.

[4] M. S. Beauchamp
Department of Neurosurgery,
University of Pennsylvania,
Philadelphia, Pennsylvania – 19104, USA.

[5] F. Vitale
Department of Physical Medicine and Rehabilitation,
University of Pennsylvania,
Philadelphia, Pennsylvania – 19104, USA.

[6] F. Vitale
Center for Neurotrauma, Neurodegeneration, and Restoration,
Corporal Michael J. Crescenz Veterans Affairs Medical Center,
Philadelphia, Pennsylvania – 19104, USA.

SUPPLEMENTARY FIGURES

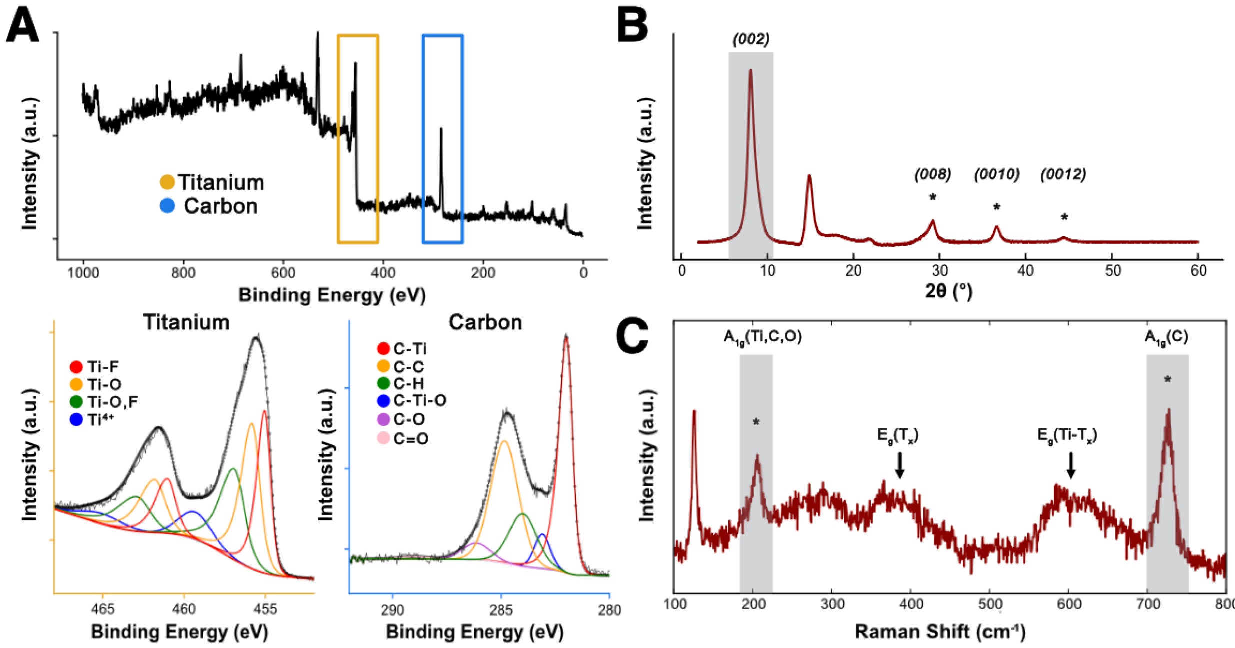

Figure S1. Structural analysis of MILD-synthesized $Ti_3C_2T_x$ MXene. (A) *Top:* XPS Survey Spectra of $Ti_3C_2T_x$ MXene, highlighting Titanium and Carbon peaks. *Bottom Left:* Core-level Ti *2p* spectra of $Ti_3C_2T_x$ MXene. *Bottom Right:* Core-level C *1s* spectra of $Ti_3C_2T_x$ MXene. **(B)** XRD spectra of $Ti_3C_2T_x$ MXene, highlighting the characteristic 002 peak. **(C)** Raman spectra of $Ti_3C_2T_x$ MXene, highlighting the characteristic out-of-plane A_{1g} peaks as well as the in-plane E_g peaks.

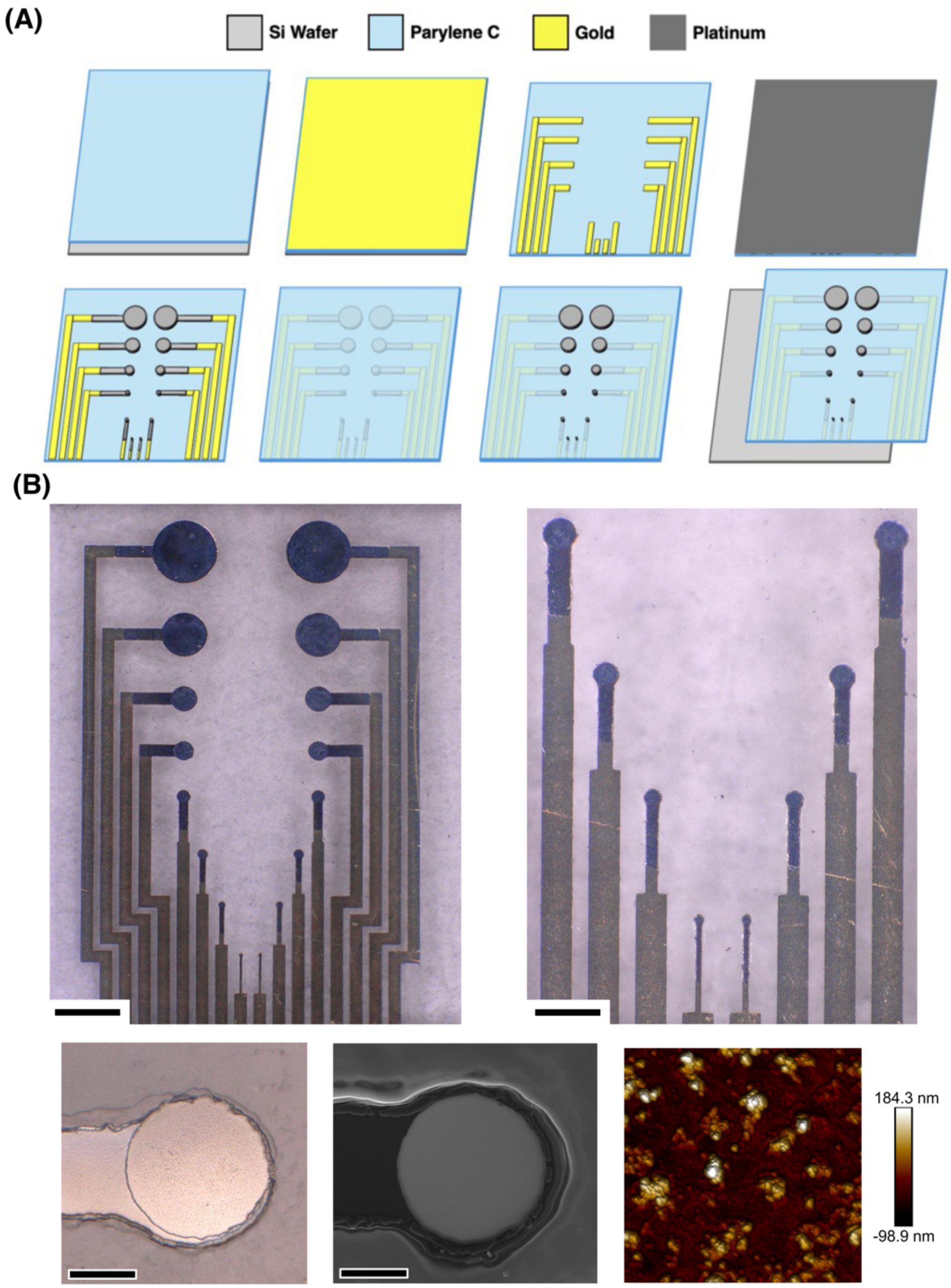

Figure S2. Fabrication schematic and images of Pt microelectrodes. (A) Schematic of the fabrication flow. **(B)** *Top:* Optical image of Pt microelectrodes (scale bar 500 μm) with zoomed-in insets of 100 μm, 75 μm, 50 μm, and 25 μm contacts. Scale bar 150 μm. *Bottom:* From left to right: optical, SEM, and AFM images of 100 μm Pt contacts. Scale bar 30 μm.

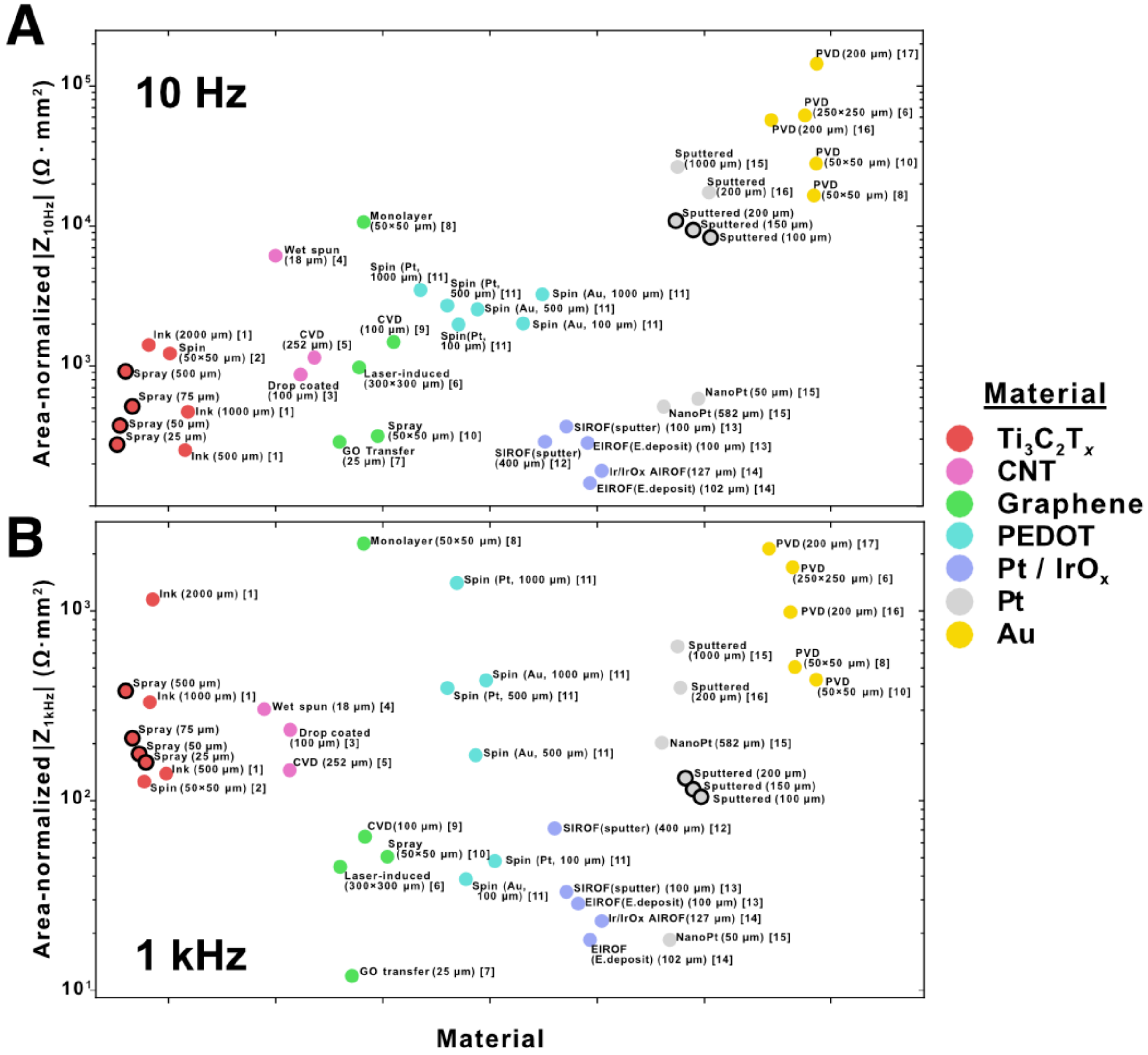

Figure S3. Area-normalized impedance across neural interface materials and electrode dimensions. Scatter plot of area-normalized impedance at **(A)** 10 Hz and **(B)** 1 kHz for $Ti_3C_2T_x$ MXene and representative neural electrode materials compiled from the literature, grouped by material class (MXene, CNT, graphene-based, PEDOT-based, Pt/IrOx, Pt, Au). Each point represents an individual device with the corresponding electrode dimension indicated. Dots with black outlines represent data from this work.[1–17]

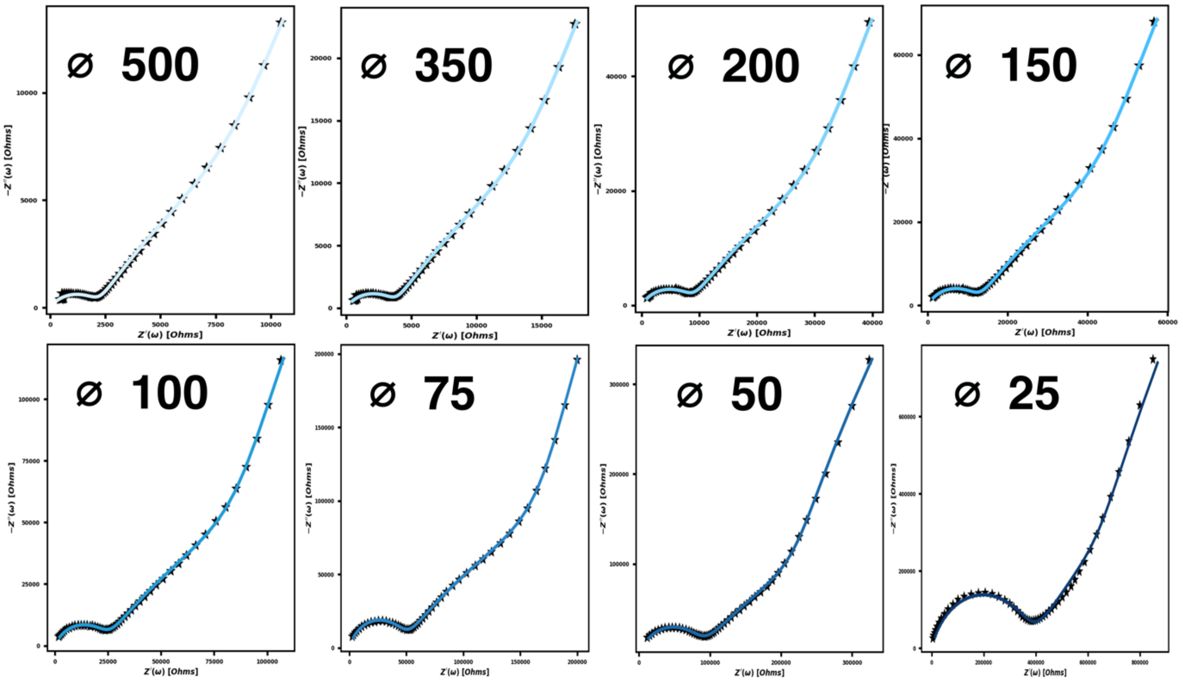

Figure S4. $Ti_3C_2T_x$ microelectrodes: Nyquist plots and equivalent circuit model fittings for all contact diameters.

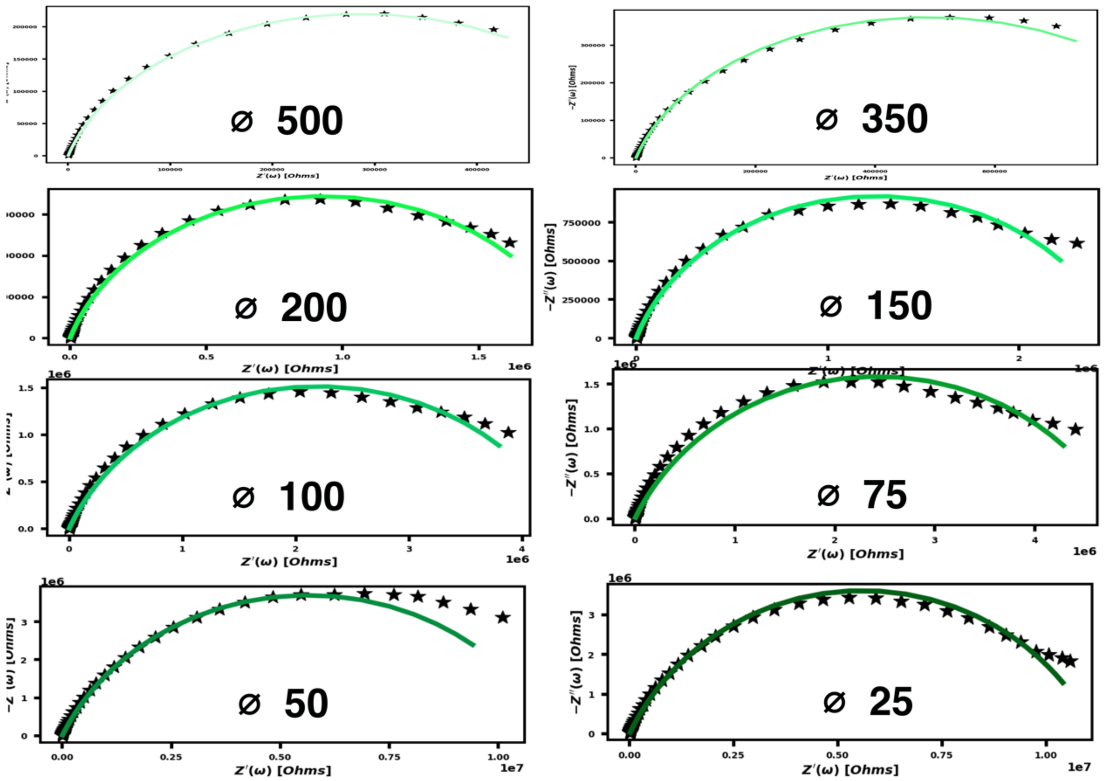

Figure S5. Pt microelectrodes: Nyquist plots and equivalent circuit model fittings for all contact diameters.

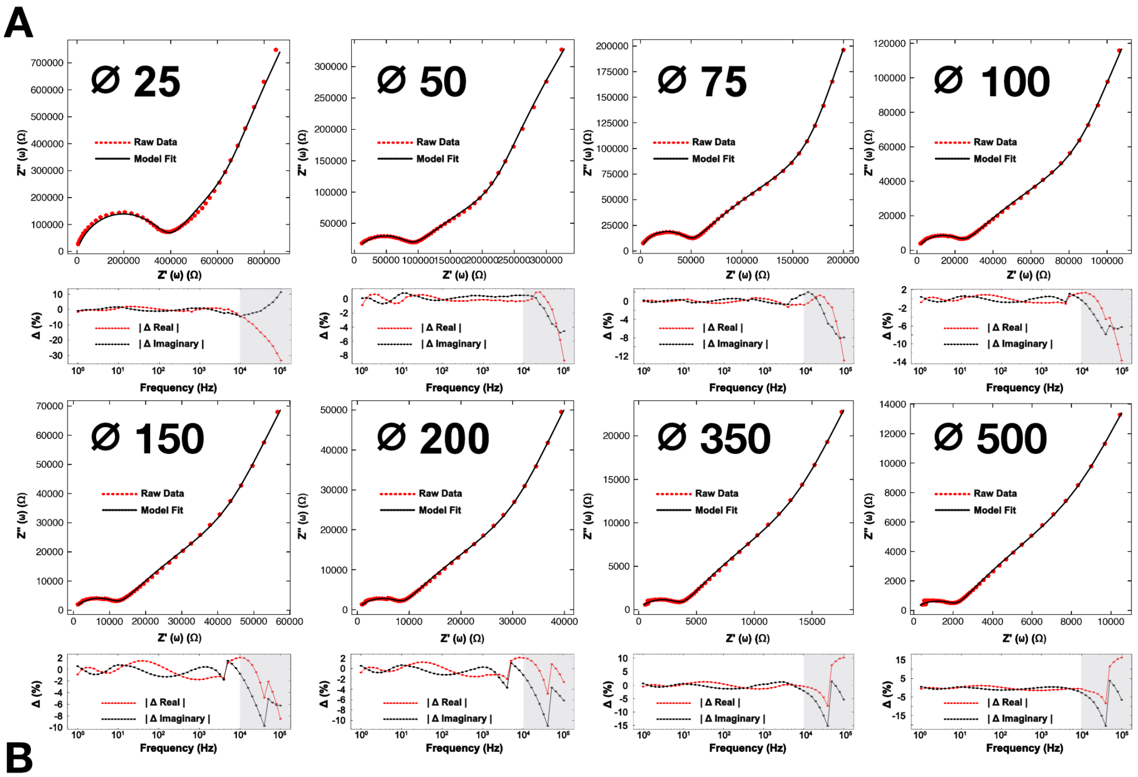

B

Electrode Diameter	25 µm	50 µm	75 µm	100 µm	150 µm	200 µm	350 µm	500 µm
RMS $Error_{Real}$ (%)	1.24	0.38	0.45	0.68	1.14	1.11	0.77	0.82
RMS % $Error_{Imaginary}$ (%)	1.39	0.41	0.47	0.51	0.77	1.00	0.85	0.80

Figure S6. Error Analysis and Model Fit Residuals. **(A)** For each contact diameter, *(Top)* Raw and fitted Nyquist plot, (*Bottom)* Real and imaginary model fit residuals across all frequencies, highlighting low error for all frequencies that satisfy KK-linearity consistency checks (1 Hz – 10 kHz). Grey-highlighted regions in residuals represent content that did not meet a KK consistency check within 2.5%. **(B)** RMS error for real and imaginary components of residuals within the KK-linear region, expressed as RMS percent error, where residuals at each frequency are normalized by the magnitude of the measured impedance $|Z_{meas}|$ for each electrode diameter.

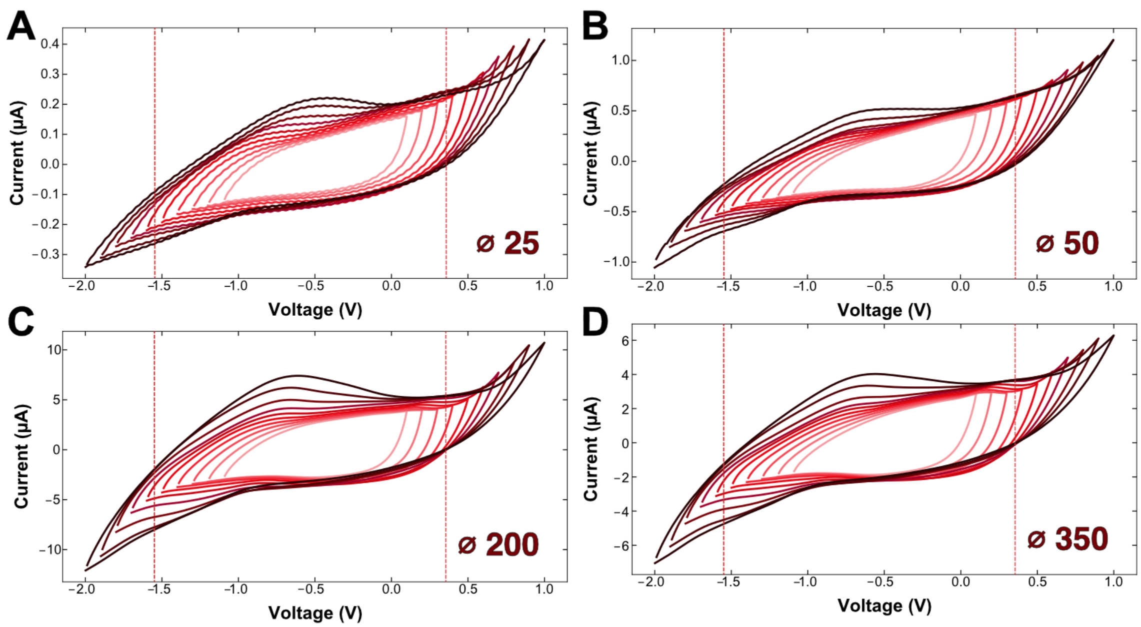

Figure S7. Water window determination for $Ti_3C_2T_x$ MXene thin-film microelectrodes. (A-D) Expanding potential windows delineating anodic and cathodic voltage limits for $Ti_3C_2T_x$ MXene thin-film microelectrodes at diameters of **(A)** 25 μm, **(B)** 50 μm, **(C)** 200 μm, and **(D)** 350 μm.

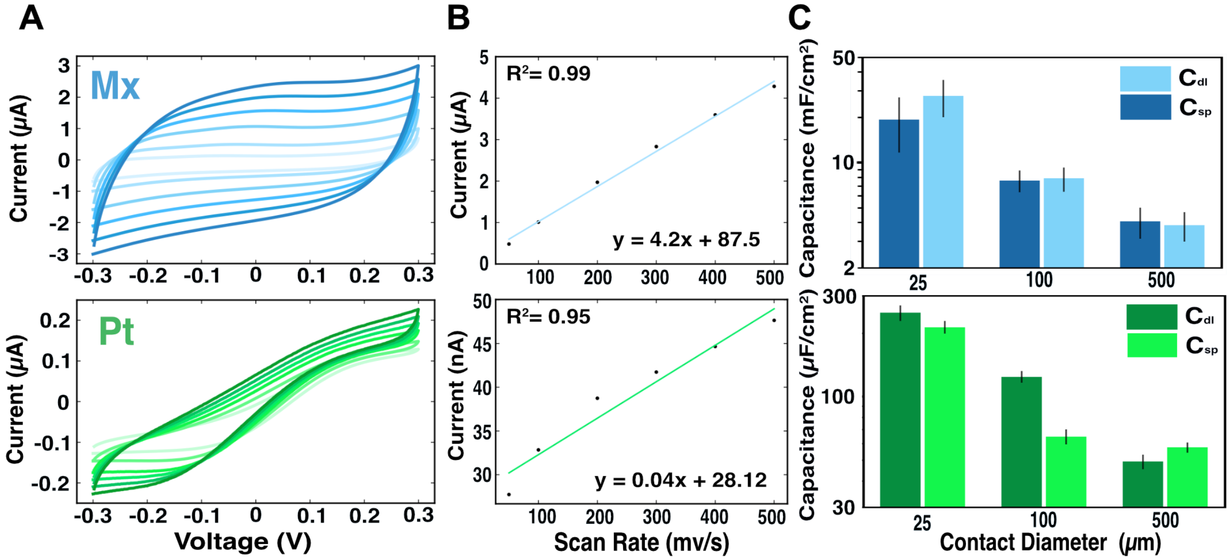

Figure S8. Specific capacitance from CVs vs C_{dl} from the equivalent circuit model. (A) Cyclic voltammograms at varying scan rates (50 mV s^{-1}, 100 mV s^{-1}, 200 mV s^{-1}, 300 mV s^{-1}, 400 mV s^{-1}, 500 mV s^{-1}) for 350 μm microelectrodes. *Top:* $Ti_3C_2T_x$, *Bottom:* Pt. **(B)** Current vs. scan rate for *Top:* $Ti_3C_2T_x$ and *Bottom:* Pt microelectrodes. **(C)** Comparison of the capacitance calculated from **(B)** and from the equivalent circuit model for *Top:* $Ti_3C_2T_x$, *Bottom:* Pt microelectrodes.

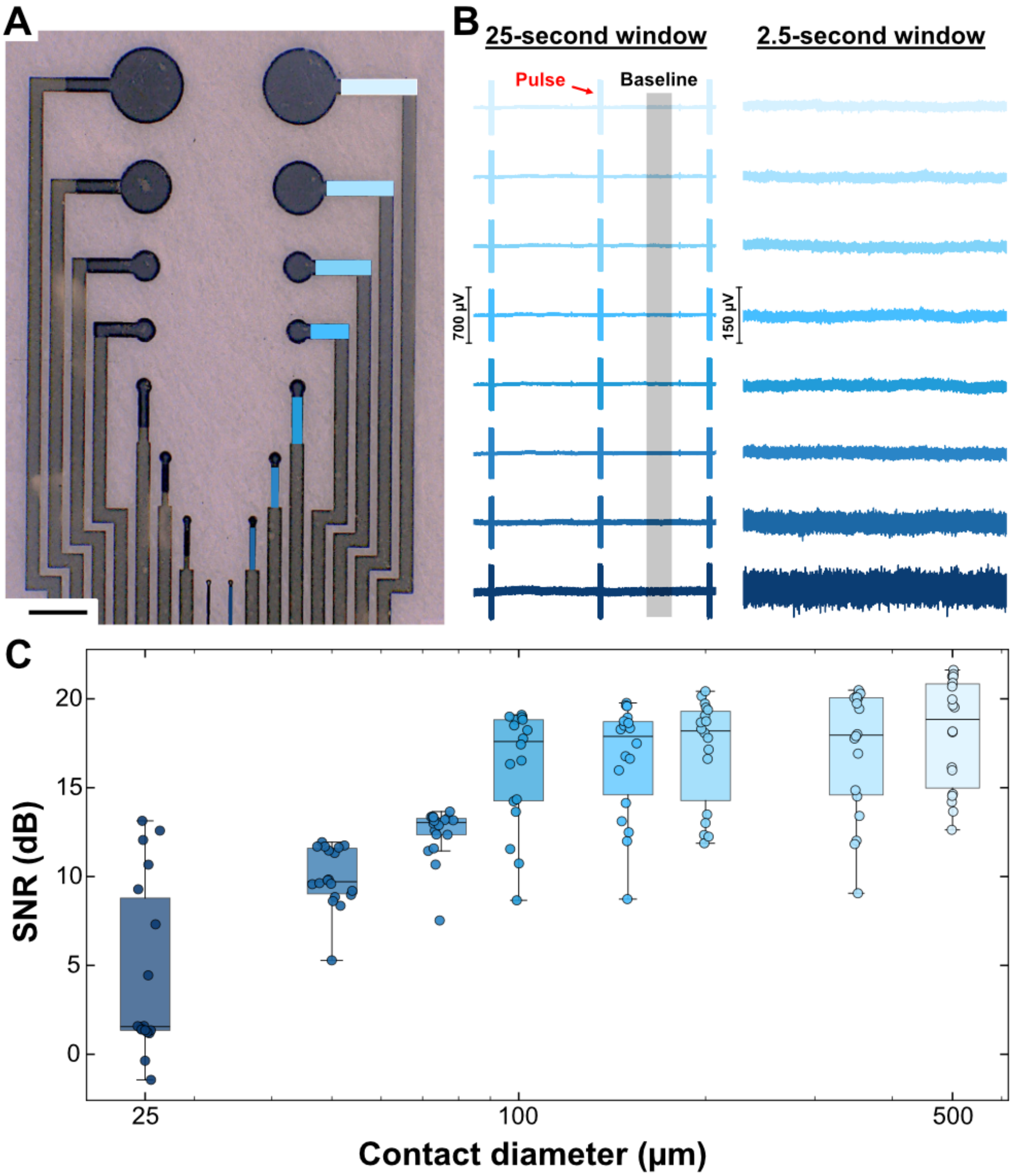

Figure S9. Relating electrode size to SNR. (A) Representative image of the $Ti_3C_2T_x$ microelectrode array. Scale bar = 500 µm. **(B)** *Left:* Three representative stimulation pulses from each electrode over a 25-second window, with the color corresponding to the electrode, on the left. *Right:* Zoomed-in 2.5-second clip of noise floor for each electrode diameter. **(C)** SNR as a function of contact diameter. N = 3 devices, 10 stim pulses per recording.

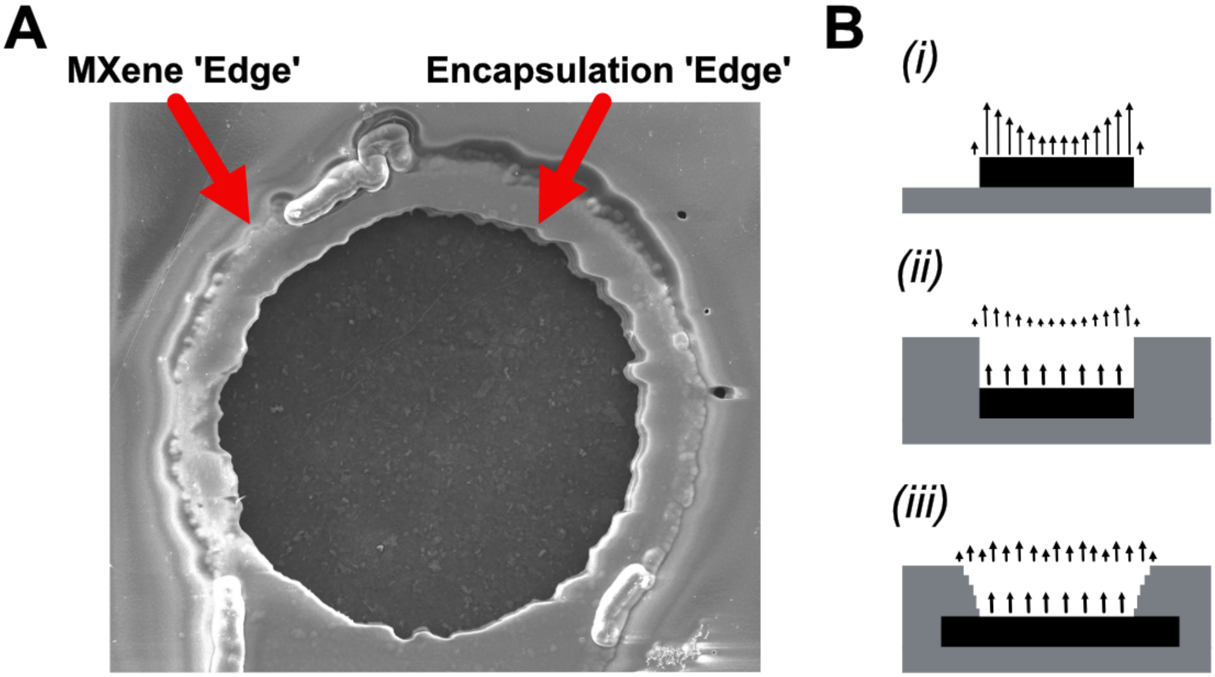

Figure S10. **$Ti_3C_2T_x$ MXene thin-film microelectrode 'edge effects' considerations. (A)** SEM image of a 100 µm diameter electrode contact. Red arrows point to the 'edge' of the spray-coated MXene, defined by the photoresist during fabrication, and the encapsulation edge, defined by the RIE etching of the parylene C encapsulation. **(B)** Expected charge distribution of electrodes fabricated with a *(i)* surface-mounted, *(ii)* simple recessed, and *(iii)* conically / irregularly recessed electrode.[18]

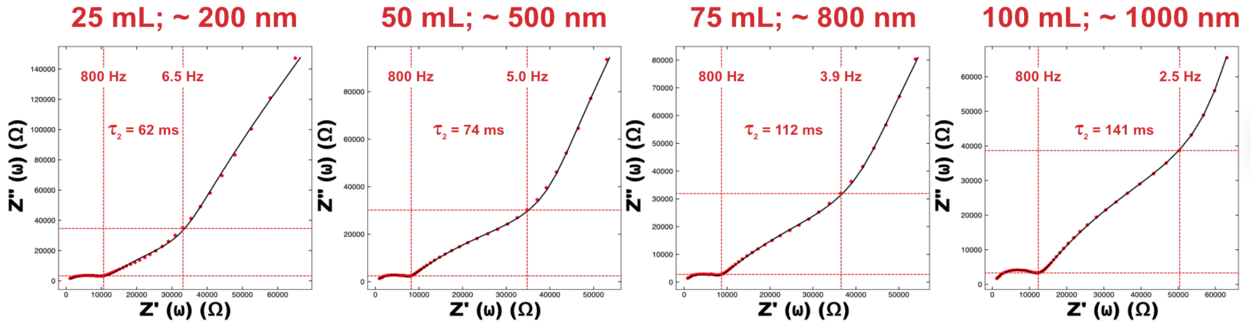

Figure S11. Emergence of low-frequency RC feature with increasing film thickness. As the thickness of the spray-coated $Ti_3C_2T_x$ MXene film increases for identically fabricated, 200 µm diameter electrodes, the frequency width and characteristic prominence of the 'bulk-layered' RC time-constant grow. N = 8 electrode samples per film thickness.

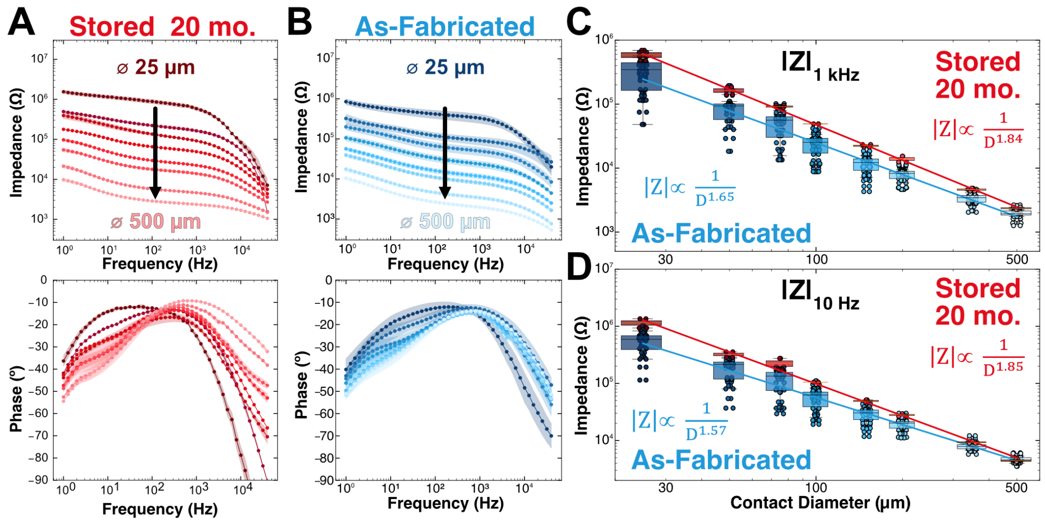

Figure S12. Shelf-life of $Ti_3C_2T_x$ MXene microelectrodes after 20 months: electrochemical impedance spectroscopy. Impedance modulus and phase spectra of $Ti_3C_2T_x$ microelectrodes of varying diameter (**A**) stored for 20 months, and (**B**) as-fabricated. Individual points represent the mean, shaded areas represent ± 1 standard deviation. (**C, D**) Impedance modulus at (**C**) 1 kHz and (**D**) 10 Hz as a function of electrode diameter.

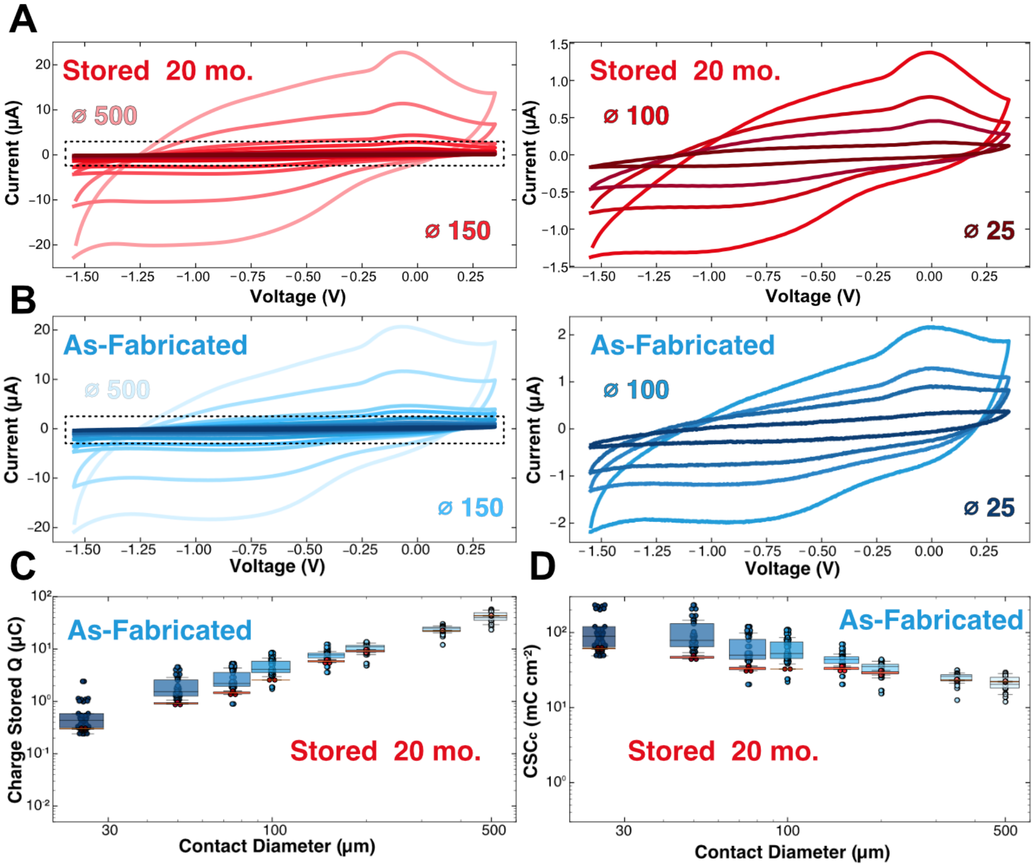

Figure S13. Shelf-life of $Ti_3C_2T_x$ MXene microelectrodes after 20 months: cyclic voltammetry and charge storage capacity. (A) *Left:* Cyclic voltammograms averaged across electrodes (n_{stored} = 4 electrodes, each size) of $Ti_3C_2T_x$ MXene microelectrodes stored for over 20

months from -1.55 V (cathodic limit) to 0.35 V (anodic limit). *Right:* Zoomed-in voltammograms for contacts ranging from 100 µm to 25 µm. **(B)** *Left:* Cyclic voltammograms of freshly fabricated $Ti_3C_2T_x$ MXene microelectrodes averaged across electrodes ($n_{as\text{-}fabricated}$ = 34 electrodes, each size) from -1.55 V (cathodic limit) to 0.35 V (anodic limit). *Right:* Zoomed-in voltammograms for contacts ranging from 100 µm to 25 µm. **(C)** Charge stored across all contact sizes for aged and as-fabricated $Ti_3C_2T_x$ devices. **(D)** Cathodic charge storage capacity across all contact sizes of aged and freshly fabricated $Ti_3C_2T_x$ devices.

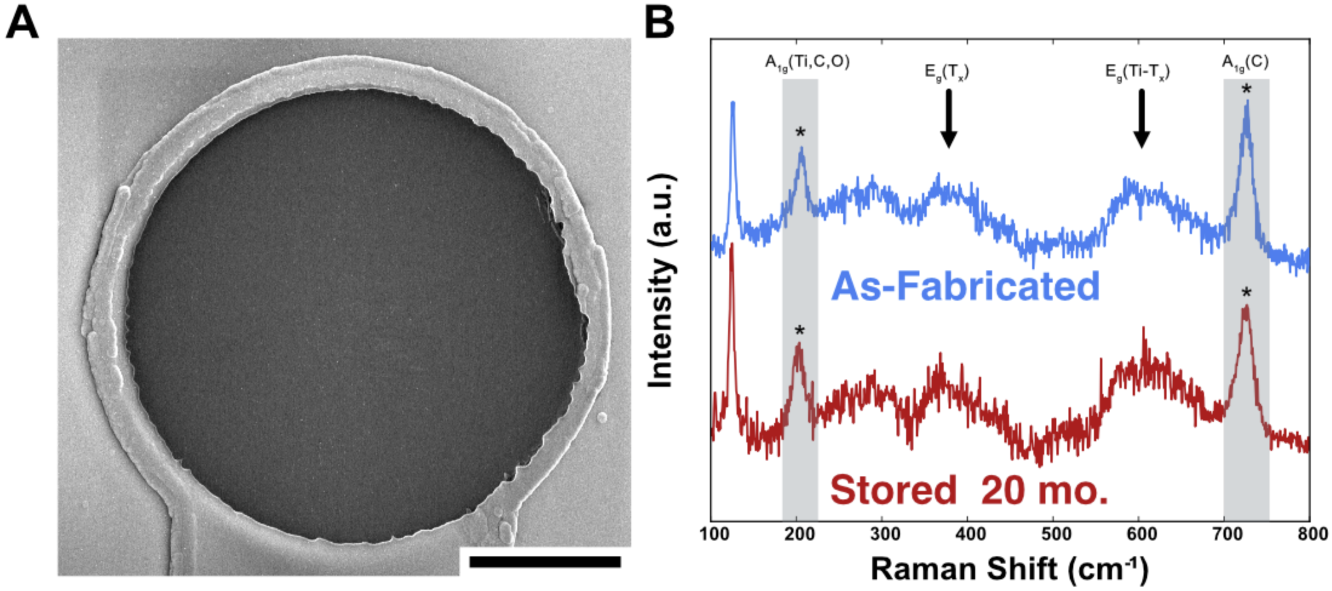

Figure S14. Shelf-life of $Ti_3C_2T_x$ MXene microelectrodes after 20 months: SEM and Raman spectroscopy. (A) SEM image of a 150 µm MXene contact on a device stored for over 20 months. Scale bar: 50 µm. **(B)** Raman spectroscopy of $Ti_3C_2T_x$ MXene microelectrodes in fresh (*blue)* and aged (*red*) devices.

SUPPLEMENTARY TABLES

Table S1. Impedance values at 1 kHz and 10 Hz for $Ti_3C_2T_x$ and Pt devices.

Contact Diameter	Frequency (Hz)	$Ti_3C_2T_x$		Platinum	
		Mean (kΩ)	S.D. (kΩ)	Mean (kΩ)	S.D. (kΩ)
500 µm	1,000	1.97	0.34	1.11	0.11
	10	4.67	0.58	74.4	12.2
350 µm	1,000	3.40	0.67	1.92	0.20
	10	8.07	1.41	145	19.6
200 µm	1,000	7.97	1.80	4.32	1.01
	10	21.2	5.72	347	201
150 µm	1,000	11.6	3.57	6.70	1.77
	10	28.3	7.98	529	277
100 µm	1,000	23.6	8.97	13.7	4.22
	10	58.2	22.7	1,100	527
75 µm	1,000	49.8	23.6	18.5	7.13
	10	117	50.8	1,390	755
50 µm	1,000	90.8	42.0	49.0	17.6
	10	188	71.9	2,670	1,170
25 µm	1,000	328	168	140	74.7
	10	550	218	4,370	1,960

Table S2. Area-normalized impedance at 10 Hz across neural interface materials and fabrication methods. Literature-reported impedance values at 10 Hz for a range of electrode materials, including $Ti_3C_2T_x$ MXene, carbon nanotubes (CNT), graphene-based systems, conductive polymers (PEDOT-based), and metal-based electrodes (Pt, Au, IrO_x).[1–17] A freely available graphical digitizer was used (plotdigitizer.com) in all instances where impedance values were extracted from plots.

Material	Deposition	Diam. (µm)	Area (mm^2)	Impedance $\|Z_{1\,kHz}\|$ (kΩ)	Area-normalized $\|Z_{1\,kHz}\|$ (Ω mm^2)	Impedance $\|Z_{10Hz}\|$ (kΩ)	Area-normalized $\|Z_{10Hz}\|$ (Ω mm^2)	Ref.
**$Ti_3C_2T_x$**	**Spray-coated**	500	0.1963	1.97	386.71	4.67	916.72	This Work
**$Ti_3C_2T_x$**	**Spray-coated**	75	0.0044	49.8	219.12	117	514.80	This Work
**$Ti_3C_2T_x$**	**Spray-coated**	50	0.0020	90.8	181.60	188	376.00	This Work
**$Ti_3C_2T_x$**	**Spray-coated**	25	0.0005	328	164.00	550	275.00	This Work
**$Ti_3C_2T_x$**	**Ink-infused**	2000	3.1416	0.369	1159.25	0.45	1413.72	[1]
**$Ti_3C_2T_x$**	**Ink-infused**	1000	0.7854	0.430	337.72	0.6	471.24	[1]
**$Ti_3C_2T_x$**	**Ink-infused**	500	0.1963	0.729	143.10	1.34	263.04	[1]
**$Ti_3C_2T_x$**	**Spin-coated**	50x50	0.0025	52.0	130.00	492.13[a]	1230.33	[2]
**CNT-$Ti_3C_2T_x$/Au**	**Drop coating**	100	0.0079	30.66[a]	242.21	110	869.00	[3]
**CNT Fiber**	**Wet-spinning**	18	0.0015	206.9[a]	310.35	4096.24[a]	6144.36	[4]
**CNT Array**	**Thermal CVD**	252	0.05	2.98[a]	149.00	22.98[a]	1149.00	[5]
**Graphene**	**Laser-induced**	300x300	0.09	0.519	46.71	10.86[a]	977.40	[6]
**Graphene/ Au**	**GO transfer**	25	0.0005	25.2	12.60	574.40[a]	287.20	[7]
**Graphene**	**Monolayer, HNO_3 doped**	50x50	0.0025	908	2270.00	4,269.18[a]	10672.95	[8]
**Fuzzy Graphene**	**Plasma enhanced CVD**	100	0.0079	8.5	67.15	188.2	1486.78	[9]
**rGO/Au**	**Spray-coated**	50x50	0.0025	21.1	52.75	126.53[a]	316.33	[10]
**PEDOT / Pt**	**Spin-coated**	500	0.1963	2.04[a]	400.45	13.15[a]	2581.35	[11]
**PEDOT / Pt**	**Spin-coated**	1000	0.7854	1.80[a]	1413.72	4.11[a]	3227.99	[11]

**PEDOT / Pt**	**Spin-coated**	100	0.0079	6.34[a]	50.09	288.73[a]	2280.97	[11]
**PEDOT / Au**	**Spin-coated**	500	0.1963	0.91[a]	178.63	12.56[a]	2465.53	[11]
**PEDOT / Au**	**Spin-coated**	1000	0.7854	0.54[a]	424.12	3.54[a]	2780.32	[11]
**PEDOT / Au**	**Spin-coated**	100	0.0079	5.10[a]	40.29	280.25[a]	2213.97	[11]
**Pt / IrO_x**	**SIROF (sputter)**	400	0.1257	0.59[a]	74.16	2.29[a]	287.85	[12]
**Pt / IrO_x**	**SIROF (sputter)**	100	0.0079	4.377	34.58	46.85[a]	370.12	[13]
**Pt / IrO_x**	**EIROF (E.deposit)**	100	0.0079	3.801	30.03	35.75[a]	282.43	[13]
**Pt / IrOx**	**EIROF (E.deposit)**	102	0.0082	2.37[a]	19.43	17.80[a]	145.96	[14]
**Ir / IrOx**	**AIROF (CV Pulse)**	127	0.0127	1.92[a]	24.38	14.06[a]	178.56	[14]
**Pt**	**NanoPt coated**	582	0.2660	0.78[a]	207.48	1.93[a]	513.38	[15]
**Pt**	**NanoPt coated**	50	0.0020	9.71[a]	19.42	270.0[a]	540.00	[15]
**Pt**	**Sputtered**	1000	0.7854	0.84[a]	659.74	33.65[a]	26428.71	[15]
**Pt**	**Sputtered**	200	0.0314	4.32	135.65	347	10895.80	This Work
**Pt**	**Sputtered**	150	0.0177	6.70	118.59	529	9363.30	This Work
**Pt**	**Sputtered**	100	0.0079	13.7	108.23	1,100	8690.00	This Work
**Pt**	**Sputtered**	200	0.0314	12.80[a]	401.92	553.90[a]	17392.46	[16]
**Au**	**PVD**	200	0.0314	67.97[a]	2134.26	4,596.96[a]	144344.54	[16]
**Au**	**PVD**	200	0.0314	31.72[a]	996.01	1821.65[a]	57199.81	[17]
**Au**	**PVD**	250x250	0.0625	27.3	1706.25	988.55[a]	61784.38	[6]
**Au**	**PVD**	50x50	0.0025	206.0	515.00	6,619.30[a]	16548.25	[8]
**Au**	**PVD**	50x50	0.0025	177.0	442.50	11,166.37[a]	27915.93	[10]

a – Values extracted from plots from the cited reference.
b – Values extracted from the image scale bar.

Table S3. Summary of all equivalent circuit model parameters for 200 µm $Ti_3C_2T_x$ and Pt microelectrodes.

Electrode	Circuit Elements	Values from Fitting	Calculated Cdl ($\mu F/cm^2$)
$Ti_3C_2T_x$	R_s (kΩ)	**0.880 ± 0.211**	
	α_0	.680 ± .014	**57.3 ± 4.31**
	Y_0 (S s^α)	(6.78 ± .061) x10^{-8}	
	R_{ct} (kΩ)	**9.15 ± 0.997**	
	R_{eq} (kΩ)	102 ± 13.7	
	W_{diff0} (kΩ)	0.276 ± .022	
	W_{diff1} (s)	(2.58 ± .103) x10^{-4}	
	α_1	.640 ± .019	**5,960 ± 982**
	Y_1 (S s^α)	(3.63 ± .161) x10^{-6}	
Platinum	R_s (kΩ)	**0.994 ± 0.153**	
	α_0	.814 ± .023	**66.9 ± 4.87**
	Y_0 (S s^α)	(3.39 ± .049) x10^{-8}	
	R_{ct} (kΩ)	**1,850 ± 236**	

Table S4. Summary of key model parameters for $Ti_3C_2T_x$ and Pt devices from EIS fitting.

Contact Diameter	Frequency (Hz)	$Ti_3C_2T_x$		Platinum		Theory
		Mean (kΩ)	S.D. (kΩ)	Mean (kΩ)	S.D. (kΩ)	
500 μm	R_s (kΩ)	0.540	.053	0.603	.055	0.720
	C_{dl} (μF/cm^2)	3,820	851	57.5	3.23	
	$R_{ct,\ surf}$ (kΩ)	2.29	.615	585	83.3	
350 μm	R_s (kΩ)	0.710	.159	.921	.185	1.03
	C_{dl} (μF/cm^2)	4,500	909	66.5	4.12	
	$R_{ct,\ surf}$ (kΩ)	3.93	.877	977	182	
200 μm	R_s (kΩ)	0.880	.211	0.994	.153	1.80
	C_{dl} (μF/cm^2)	6,020	1,031	66.9	4.87	
	$R_{ct,\ surf}$ (kΩ)	9.15	0.997	1,850	236	
150 μm	R_s (kΩ)	1.20	.346	1.64	.349	2.40
	C_{dl} (μF/cm^2)	7,830	1,440	64.7	5.38	
	$R_{ct,\ surf}$ (kΩ)	13.1	1.54	2,580	610	
100 μm	R_s (kΩ)	1.72	.641	2.45	.682	3.60
	C_{dl} (μF/cm^2)	8,470	1,970	83.0	8.94	
	$R_{ct,\ surf}$ (kΩ)	25.4	6.78	4,390	855	
75 μm	R_s (kΩ)	2.41	.950	3.26	1.13	4.80
	C_{dl} (μF/cm^2)	8,520	2,100	102	9.03	
	$R_{ct,\ surf}$ (kΩ)	49.4	8.49	4,840	970	
50 μm	R_s (kΩ)	4.02	1.23	5.16	1.42	7.20
	C_{dl} (μF/cm^2)	14,300	4,580	145	11.62	
	$R_{ct,\ surf}$ (kΩ)	89.9	10.2	10,200	1,185	
25 μm	R_s (kΩ)	8.35	1.84	6.06	1.21	14.40
	C_{dl} (μF/cm^2)	27,600	7,590	213	14.72	
	$R_{ct,\ surf}$ (kΩ)	381	40.9	11,200	1,460	

Table S5. Charge and cathodic charge storage capacity (CSC_c) for $Ti_3C_2T_x$ and Pt microelectrodes.

Contact Diameter	$Ti_3C_2T_x$		Platinum	
	Charge (μC)	CSC (mC/cm^2)	Charge (μC)	CSC (mC/cm^2)
**500 μm**	40.8 ± 11.4	20.8 ± 5.83	1.31 ± 0.105	0.669 ± 0.054
**350 μm**	23.9 ± 3.74	24.8 ± 3.89	0.671 ± 0.056	0.698 ± 0.058
**200 μm**	10.2 ± 2.96	32.5 ± 9.44	0.247 ± 0.053	0.786 ± 0.170
**150 μm**	7.67 ± 1.68	43.4 ± 9.49	0.164 ± 0.050	0.925 ± 0.281
**100 μm**	4.69 ± 1.79	59.7 ± 22.8	0.073 ± 0.008	0.936 ± 0.104
**75 μm**	2.72 ± 1.18	61.5 ± 26.6	0.045 ± 0.005	1.02 ± 0.106
**50 μm**	1.87 ± 1.06	95.2 ± 53.8	0.037 ± 0.019	1.87 ± 0.955
**25 μm**	0.588 ± 0.48	119.9 ± 97.3	0.021 ± 0.015	4.29 ± 2.99

Table S6. Capacitances calculated from C_{dl} in the impedance equivalent circuit model and specific capacitance from CV for $Ti_3C_2T_x$ and Pt microelectrodes.

Contact Diameter	$Ti_3C_2T_x$		Platinum	
	Double Layer Capacitance (μF/cm^2)	Specific Capacitance (μF/cm^2)	Double Layer Capacitance (μF/cm^2)	Specific Capacitance (μF/cm^2)
**500 μm**	3820 ± 851	4060 ± 942	57.5 ± 3.23	49.2 ± 3.85
**350 μm**	4500 ± 909	4390 ± 892	66.5 ± 4.12	46.0 ± 4.05
**200 μm**	6020 ± 1031	5550 ± 1180	66.9 ± 4.87	47.5 ± 3.38
**150 μm**	7830 ± 1440	7590 ± 1240	64.7 ± 5.38	124 ± 7.69
**100 μm**	8470 ± 1970	8760 ± 1960	83.0 ± 8.94	113 ± 9.92
**75 μm**	8520 ± 2100	8930 ± 2390	102 ± 9.03	140 ± 13.4
**50 μm**	14300 ± 4580	13100 ± 4130	145 ± 11.62	381 ± 53.7
**25 μm**	27600 ± 7590	19300 ± 7640	213 ± 14.72	249 ± 21.1

Table S7. Comparative electrochemical parameters of $Ti_3C_2T_x$, sputtered Pt, gold, PEDOT: PSS / Pt, Sputtered Pt, SIROF, PtIr, and CNT microelectrodes across the microscale from 500 µm to 18 µm.

	Diam. (µm)	1 kHz |Z| (kΩ)	10 Hz |Z| (kΩ)	R_s (kΩ)	CSC_c (mC cm^{-2})	$Curr_{max}$ (µA)	CIC_c (µC cm^{-2})	Ref.
$Ti_3C_2T_x$	500	1.97	4.67	0.54	20.8	1880	480	This Work
	150	11.6	28.3	1.20	43.4	231	654	
	100	23.6	58.2	1.72	59.7	97.3	619	
	50	90.8	188	4.02	95.2	30.6	779	
Sputtered Pt	500	1.11	74.4	0.603	0.669	134	34.0	This Work
	150	6.70	529	1.64	0.925	33.6	95.0	
	100	13.7	1,100	2.45	0.936	22.9	146	
	50	49.0	2,670	5.16	1.87	5.60	144	
Gold	500	4.5[a]	300[a]	0.8[a]	0.1[a,b]	300[a,c]	200 [a,b,c]	[11,19]
	150	70[a]	5,000[a]	2.5[a]	0.5[a,b]	30[a,c]	200 [a,b,c]	
	100	170[a]	10,500[a]	4.2[a]	0.8[a,b]	12[a,c]	200 [a,b,c]	
	50	500[a]	30,500[a]	8.5[a]	1[a,b]	3[a,c]	200[a,b,c]	
PEDOT: PSS / Pt	500	2[a]	10.5[a]	1.5[a]	3.5[a,b]	850[a,c]	650[a,b,c]	[11,19]
	150	4.5[a]	190[a]	2.5[a]	5[a,b]	350[a,c]	2700[a,b,c]	
	100	6[a]	300[a]	5.2[a]	5[a,b]	150[a,c]	2700[a,b,c]	
	50	15[a]	1,000[a]	9.5[a]	6[a,b]	40[a,c]	2700[a,b,c]	
Sputtered Pt	500	8[a]	300[a]	1.5[a]	5.1[a,b]	750[a,c]	500 [a,b,c]	[11,19]
	150	75[a]	3,000[a]	2.5[a]	6.0[a,b]	100[a,c]	750 [a,b,c]	
	100	200[a]	5,000[a]	4.2[a]	6.2[a,b]	50[a,c]	850 [a,b,c]	
	50	450[a]	10,500[a]	9.5[a]	8.2[a,b]	10[a,c]	850 [a,b,c]	
SIROF	400	0.501[a]	2.51[a]	N/A	133	5,966[d]	1,900	[12]
PtIr Wire	18	1,100[a]	60,000[a]	N/A	1.2	42.5[d]	150	[4]
CNT Fiber	18	200[a]	4,000[a]	N/A	372	1,570[d]	6,520	[4]

a – Values extracted from plots from the cited references.

b – Roughly doubled value due to cathodic + anodic normalization.

c – Values obtained with positive voltage bias during measurements.

d – Values calculated from reported pulse-width, CIC, and surface area.

Table S8. Max current and cathodic charge injection capacity of $Ti_3C_2T_x$ microelectrodes at varying pulse width.

$Ti_3C_2T_x$ MXene							
Contact Size	Parameter	100 µs	250 µs	500 µs	750 µs	1000 µs	1500 µs
500 µm	Current (µA)	3882.2 ± 540.4	2976.8 ± 321.4 µA	1884.1 ± 200.1	1436.8 ± 151.5	1598.8 ± 31.7	
	CIC (µC/cm^2)	197.7 ± 27.5	379.0 ± 40.9	479.7 ± 50.9	548.8 ± 57.8	814.2 ± 16.1	
350 µm	Current (µA)	2504.0 ± 253.3	1638.4 ± 200.0	891.9 ± 128.7	679.2 ± 48.9	1629.4 ± 49.1	
	CIC (µC/cm^2)	260.2 ± 26.3	425.7 ± 52.0	463.5 ± 66.9	529.5 ± 38.1	1693.6 ± 51.0	
200 µm	Current (µA)	673.1 ± 86.2	545.0 ± 91.6	364.9 ± 51.2	313.6 ± 51.5	266.8 ± 36.1	219.2 ± 29.7
	CIC (µC/cm^2)	214.2 ± 27.4	433.7 ± 72.9	580.8 ± 81.5	748.7 ± 123.1	849.4 ± 115.0	1046.9 ± 142.2
150 µm	Current (µA)	497.7 ± 2.05	368.5 ± 70.8	231.1 ± 29.5	196.3 ± 19.2	177.5 ± 14.6	147.5 ± 9.01
	CIC (µC/cm^2)	281.6 ± 1.16	521.4 ± 100.1	653.8 ± 83.4	833.5 ± 81.9	1004.5 ± 83.1	1252.1 ± 76.5
100 µm	Current (µA)	282.2 ± 36.5	180.0 ± 63.3	97.3 ± 15.8	103.2 ± 7.3	94.7 ± 7.1	
	CIC (µC/cm^2)	359.3 ± 46.5	573.0 ± 201.6	619.4 ± 100.8	986.0 ± 70.3	1205.9 ± 90.8	
75 µm	Current (µA)	186.0 ± 31.9	116.3 ± 52.8	59.0 ± 18.8	48.4 ± 11.3	47.3 ± 15.6	
	CIC (µC/cm^2)	421.08 ± 72.3	658.1 ± 299.1	668.1 ± 213.3	823.2 ± 192.1	1072.8 ± 355.3	
50 µm	Current (µA)	60.3 ± 10.7	45.9 ± 9.003311	30.6 ± 4.18	24.9 ± 5.3	21.5 ± 2.9	
	CIC (µC/cm^2)	307.2 ± 54.9	584.6 ± 114.6	779.2 ± 106.5	952.4 ± 204.6	1096.3± 150.0	
25 µm	Current (µA)	11.9 ± 4.1	18.5 ± 12.1	4.88 ± 1.9	4.2 ± 2.38	5.7 ± 2.8	
	CIC (µC/cm^2)	243.8 ± 83.6	943.4 ± 616.3	497.0 ± 195.5	650.2 ± 364.5	1172.9 ± 574.2	

Table S9. Max current and cathodic charge injection capacity of sputtered Pt microelectrodes at varying pulse width.

Sputtered Platinum							
Contact Size	Parameter	100 µs	250 µs	500 µs	750 µs	1000 µs	1500 µs
500 µm	Current (µA)	623.7 ± 69.7	364.3 ± 51.7	133.7 ± 8.35	426.8 ± 19.2	726.7 ± 44.5	
	CIC (µC/cm^2)	31.7 ± 3.5	46.3 ± 6.5	34.0 ± 2.12	163.0 ± 7.3	388.7 ± 79.1	
350 µm	Current (µA)	472.9 ± 24.8	440.6 ± 118.1	101.6 ± 3.0	397.9 ± 1.88	626.8 ± 44.4	
	CIC (µC/cm^2)	49.1 ± 2.58	114.4 ± 30.7	52.8 ± 1.58	310.2 ± 1.47	634.2 ± 73.7	
200 µm	Current (µA)	122.0 ± 19.5	142.1 ± 51.3	67.6 ± 15.9	74.5 ± 2.59	76.5 ± 2.91	75.6 ± 2.83
	CIC (µC/cm^2)	38.8 ± 6.21	113.1 ± 40.9	107.6 ± 25.3	178.0 ± 6.20	243.6 ± 9.29	361.1 ± 13.5
150 µm	Current (µA)	89.2 ± 14.1	102.1 ± 26.7	33.6 ± 2.84	74.4 ± 2.75	74.3 ± 2.52	76.3 ± 3.46
	CIC (µC/cm^2)	50.5 ± 7.98	144.4 ± 37.9	95.0 ± 8.04	315.9 ± 11.7	420.6 ± 14.1	648.1 ± 11.0
100 µm	Current (µA)	37.6 ± 8.8	27.488 ± 13.7	22.9 ± 8.06	21.35 ± 8.09	18.0 ± 2.64	
	CIC (µC/cm^2)	47.9 ± 11.2	87.3 ± 43.6	146.0 ± 51.3	203.8 ± 77.3	229.8 ± 33.7	
75 µm	Current (µA)	29.1 ± 11.6	23.6 ± 9.4	15.0 ± 4.21	14.3 ± 0.28	18.2 ± 4.3	
	CIC (µC/cm^2)	65.9 ± 26.3	133.6 ± 53.4	170.2 ± 47.7	243.32 ± 4.8	413.0 ± 97.5	
50 µm	Current (µA)	29.1 ± 19.6	8.3 ± 4.8	5.6 ± 2.9	4.6 ± 2.4	4.3 ± 1.38	
	CIC (µC/cm^2)	148.4 ± 100.2	106.1 ± 61.3	143.9 ± 74.4	176.9 ± 95.2	221.5 ± 70.4	
25 µm	Current (µA)	4.83 ± 0.93	2.76 ± 1.03	1.38 ± 0.55	1.64 ± 0.61	3.11 ± 2.09	
	CIC (µC/cm^2)	98.1 ± 19.0	140.7 ± 52.6	140.8 ± 56.5	250.6 ± 93.4	635.0 ± 209.1	

Table S10. Tentative estimates of minimum electrode size based on representative recording and stimulation requirements. Model equations arising from impedance fitting and CIC values with representative stimulation amplitudes set as magnitude guidelines from other bodies of work.[20–24] Equations from this table are derived from the impedance fit calculation from Figure 2, as well as from the calculation of CIC in Figure 5. Both equations and their corresponding variables are provided below in supplementary equations.

	**Recording** $D = \left(\frac{\lvert Z\rvert}{e^{b}}\right)^{1/m}$		**Stimulation** $D = 2\sqrt{\frac{A_{\min}\,pw}{\pi\,CIC}}$		
**Condition**	Local Fields ($\lvert Z_{10\ Hz}\rvert$ > 1 MΩ)	Multi-Unit Activity ($\lvert Z_{1\ kHz}\rvert$ > 1 MΩ)	Mouse Model A_{min} = 5 μA	Swine Model A_{min} = 50 μA	Human Model A_{min} = 100 μA
**MXene**	*m= -1.57, b= 18.19* **D = 16 μm**	*m= -1.65, b= 17.73* **D = 11 μm**	*pw=500, CIC=592* **D = 23 μm**	*pw=500, CIC=592* **D = 73 μm**	*pw=500, CIC=592* **D = 104 μm**
**Platinum**	*m= -1.39, b= 19.93* **D = 81 μm**	*m= -1.57, b= 16.69* **D = 6 μm**	*pw= 500, CIC=111* **D = 54 μm**	*pw=500, CIC=111* **D = 169 μm**	*pw=500, CIC=111* **D = 240 μm**

Table S11. Influence of processing parameters on the performance of $Ti_3C_2T_x$ MXene microelectrodes.[1,2,25–27]

**Contact Scale**	**MXene Prep**	**Diameter (μm)**	**Area (mm^2)**	**Impedance $\lvert Z_{10Hz}\rvert$ (kΩ)**	**Area-normalized $\lvert Z_{10Hz}\rvert$ (Ω mm^2)**	**Ref.**
**MICRO SCALE**	Thick spray-coat	75	0.00442	117	514.8	This Work
	Thin spray-coat	50x50	0.00250	5,000[a]	12,500.0	[2]
	Spin-coat	50x50	0.00250	500[a]	1250.0	[26]
	Dip-coat	100	0.00785	100[a]	785.4	[27]
**MACRO SCALE**	Thick spray-coat	3,000	7.06858	0.350[a]	2,474.2	[25]
	Thin spray-coat	3,000	7.06858	0.750[a]	5,301.8	[25]
	Blade-coat	3,000	7.06858	0.300[a]	2,120.7	[25]
	MXtrode	3,000	7.06858	0.2414	1,704.3	[1]

a – Values extracted from plots in the cited reference.

SUPPLEMENTARY EQUATIONS

Equation 1: Diameter scaling with impedance. Where |Z| is the impedance limit of interest, m, and b are derived from the impedance fit, calculated in Figure 2.

$$D = \left(\frac{|Z|}{e^{b}}\right)^{1/m}$$

Equation 2: Diameter scaling with charge injection capacity. Where A_{min} is the minimum current amplitude of interest, *pw* is the pulse-width of the cathodic current pulse, and *CIC* is the charge injection capacity for that material, calculated and provided in Figure 5 for both MXene and Platinum.

$$D = 2\sqrt{\frac{A_{\min} \cdot pw}{\pi CIC}}$$

REFERENCES

1. Driscoll, N. *et al.* MXene-infused bioelectronic interfaces for multiscale electrophysiology and stimulation. *Sci. Transl. Med.* **13**, eabf8629 (2021).
2. Driscoll, N. *et al.* Two-Dimensional Ti3C2 MXene for High-Resolution Neural Interfaces. *ACS Nano* (2018).
3. Cheng, L. *et al.* Three-dimensional MXene/carbon nanotube composite electrodes in flexible 64-channel arrays for noninvasive electromyography signal acquisition. *Sci. China Mater.* **67**, 2977–2984 (2024).
4. Vitale, F., Summerson, S. R., Aazhang, B., Kemere, C. & Pasquali, M. Neural Stimulation and Recording with Bidirectional, Soft Carbon Nanotube Fiber Microelectrodes. *ACS Nano* **9**, 4465–4474 (2015).
5. Wang, K., Fishman, H. A., Dai, H. & Harris, J. S. Neural Stimulation with a Carbon Nanotube Microelectrode Array. *Nano Lett.* **6**, 2043–2048 (2006).
6. Lu, Y., Lyu, H., Richardson, A. G., Lucas, T. H. & Kuzum, D. Flexible Neural Electrode Array Based-on Porous Graphene for Cortical Microstimulation and Sensing. *Sci Rep* **6**, 33526 (2016).
7. Viana, D. *et al.* Nanoporous graphene-based thin-film microelectrodes for in vivo high-resolution neural recording and stimulation. *Nat. Nanotechnol.* **19**, 514–523 (2024).
8. Driscoll, N. *et al.* Multimodal in vivo recording using transparent graphene microelectrodes illuminates spatiotemporal seizure dynamics at the microscale. *Commun Biol* **4**, 136 (2021).
9. Garg, R. *et al.* Graphene and Poly(3,4-ethylenedioxythiophene)–Polystyrene Sulfonate Hybrid Nanostructures for Input/Output Bioelectronics. *ACS Appl. Nano Mater.* **6**, 8495–8505 (2023).
10. Murphy, B. *et al.* Vitamin C-Reduced Graphene Oxide Coatings Improve the Performance and Stability of Multimodal Microelectrodes for Neural Recording, Stimulation,

and Dopamine Sensing. *SSRN Journal* https://doi.org/10.2139/ssrn.4086945 (2022) doi:10.2139/ssrn.4086945.

11. Ganji, M. *et al.* Scaling Effects on the Electrochemical Performance of poly(3,4-ethylenedioxythiophene (PEDOT), Au, and Pt for Electrocorticography Recording. *Adv. Funct. Mater.* (2017).
12. Cogan, S. F. *et al.* Sputtered iridium oxide films for neural stimulation electrodes. *J Biomed Mater Res* **89B**, 353–361 (2009).
13. Kang, X.-Y. *et al.* Fabrication and electrochemical comparison of SIROF-AIROF-EIROF microelectrodes for neural interfaces. in *2014 36th Annual International Conference of the IEEE Engineering in Medicine and Biology Society* 478–481 (2014). doi:10.1109/EMBC.2014.6943632.
14. Meyer, R. D., Cogan, S. F., Nguyen, T. H. & Rauh, R. D. Electrodeposited iridium oxide for neural stimulation and recording electrodes. *IEEE Trans. Neural Syst. Rehabil. Eng.* **9**, 2–11 (2001).
15. Boehler, C., Carli, S., Fadiga, L., Stieglitz, T. & Asplund, M. Tutorial: guidelines for standardized performance tests for electrodes intended for neural interfaces and bioelectronics. *Nat Protoc* **15**, 3557–3578 (2020).
16. Fan, B., Wolfrum, B. & Robinson, J. T. Impedance scaling for gold and platinum microelectrodes. *J. Neural Eng.* (2021).
17. Apollo, N. V. *et al.* Soft, Flexible Freestanding Neural Stimulation and Recording Electrodes Fabricated from Reduced Graphene Oxide. *Adv Funct Materials* **25**, 3551–3559 (2015).
18. Suesserman, M. F., Spelman, F. A. & Rubinstein, J. T. In vitro measurement and characterization of current density profiles produced by nonrecessed, simple recessed, and radially varying recessed stimulating electrodes. *IEEE Transactions on Biomedical Engineering* **38**, 401–408 (1991).

19. Ganji, M., Tanaka, A., Gilja, V., Halgren, E. & Dayeh, S. A. Scaling Effects on the Electrochemical Stimulation Performance of Au, Pt, and PEDOT:PSS Electrocorticography Arrays. *Adv. Funct. Mater.* (2017).

20. Lycke, R. *et al.* Low-threshold, high-resolution, chronically stable intracortical microstimulation by ultraflexible electrodes. *Cell Rep* **42**, 112554 (2023).

21. Cogan, S. F., Ludwig, K. A., Welle, C. G. & Takmakov, P. Tissue damage thresholds during therapeutic electrical stimulation. *J Neural Eng* **13**, 021001 (2016).

22. Urdaneta, M. E. *et al.* The Long-Term Stability of Intracortical Microstimulation and the Foreign Body Response Are Layer Dependent. *Front. Neurosci.* **16**, (2022).

23. Kim, W. J. *et al.* Superior cervical ganglion stimulation results in potent cerebral vasoconstriction in swine. *J Vasc Interv Neurol* **13**, 35–41 (2022).

24. Hughes, C. L. *et al.* Perception of microstimulation frequency in human somatosensory cortex. *eLife* **10**, e65128 (2021).

25. Shankar, S. *et al.* Effect of the deposition process on the stability of $Ti_3 C_2 T_x$ MXene films for bioelectronics. *2D Mater.* **10**, 044001 (2023).

26. Shankar, S. *et al.* Transparent MXene Microelectrode Arrays for Multimodal Mapping of Neural Dynamics. *Adv Healthcare Materials* **14**, 2402576 (2025).

27. Bi, L. *et al.* Soft, Multifunctional MXene-Coated Fiber Microelectrodes for Biointerfacing.
